# Brief Overview of a Decade of Genome-Wide Association Studies on Primary Hypertension

**DOI:** 10.1155/2018/7259704

**Published:** 2018-01-30

**Authors:** Afifah Binti Azam, Elena Aisha Binti Azizan

**Affiliations:** Department of Medicine, The National University of Malaysia Medical Centre, Kuala Lumpur, Malaysia

## Abstract

Primary hypertension is widely believed to be a complex polygenic disorder with the manifestation influenced by the interactions of genomic and environmental factors making identification of susceptibility genes a major challenge. With major advancement in high-throughput genotyping technology, genome-wide association study (GWAS) has become a powerful tool for researchers studying genetically complex diseases. GWASs work through revealing links between DNA sequence variation and a disease or trait with biomedical importance. The human genome is a very long DNA sequence which consists of billions of nucleotides arranged in a unique way. A single base-pair change in the DNA sequence is known as a single nucleotide polymorphism (SNP). With the help of modern genotyping techniques such as chip-based genotyping arrays, thousands of SNPs can be genotyped easily. Large-scale GWASs, in which more than half a million of common SNPs are genotyped and analyzed for disease association in hundreds of thousands of cases and controls, have been broadly successful in identifying SNPs associated with heart diseases, diabetes, autoimmune diseases, and psychiatric disorders. It is however still debatable whether GWAS is the best approach for hypertension. The following is a brief overview on the outcomes of a decade of GWASs on primary hypertension.

## 1. Introduction

Hypertension is highly prevalent globally. The estimated number of people with uncontrolled hypertension is nearly 1 billion (around 15% of the world population), with the number predicted to increase to 1.56 billion by the year 2025 [1]. Due to its high prevalence, hypertension is the leading risk factor for cardiovascular disease, stroke, and end-stage kidney diseases. The increased risk of cardiovascular mortality and morbidity has led to the estimation that hypertension causes 13% of all deaths (around 7.5 million deaths worldwide) [2]. Patients are considered to have hypertension when their systolic blood pressure is ≥140 mmHg and/or their diastolic blood pressure is ≥90 mmHg [3]. However, raised blood pressure, even within the normal range, is positively and continuously related to mortality and morbidity—each increment of 20 (systolic)/10 (diastolic) mmHg of blood pressure doubles the risk of cardiovascular diseases [2]. Hence, the number of people at risk is higher as the prevalence of raised blood pressure for adults (aged ≥25 years) is around 40% [2].

The majority of hypertension in the general population occur idiopathically with no apparent causes and therefore are categorized as primary hypertension. The remaining hypertensive cases (about 5%) are categorized as secondary hypertension as the raised blood pressure occur secondary to other causes/diseases, for example, hypertension due to aldosteronism, pheochromocytoma, renovascular diseases, or even Mendelian forms of hypertension [4, 5]. However, despite being classified as having no apparent cause, studies of familial aggregation on primary hypertensive patients have found associations of blood pressure among siblings and between parents and children, indicating that genetic factors contribute to the high blood pressure among primary hypertensive patients. Genetic factors have been estimated to explain 30–50% of the interindividual variation in blood pressure which significantly predisposes family (siblings/children) of primary hypertensive patients to hypertension [6]. These heritable genetic factors, in addition to environment and demographic factors, play a major role in interindividual variation in blood pressure [7]. Therefore, extensive genetic research has been conducted over the years, including genome-wide association studies (GWASs), to help elucidate primary hypertension's heritability.

## 2. Outcomes of Genome-Wide Association Studies on Primary Hypertension

GWASs have identified over three hundred plus SNPs/loci associated with blood pressure and/or primary hypertension over the past decade (Table 1). Meta-analyses of GWASs have made the biggest contribution as they allowed for larger sample sizes and more extensive imputation panels. Despite these advancements, genetic variation identified so far only explains ~3–6% of the variance for blood pressure, approximately 1 mmHg per allele systolic blood pressure or 0.5 mmHg per allele diastolic blood pressure [8–12]. Further, the vast majority of GWASs were performed predominantly in Caucasian populations with only a few studies assessing or replicating in other populations even though high blood pressure burden risk is ranked number one in Southeast Asia, Central Asia, North Africa, and Middle East [13–40]. This suggests the existence of many more undiscovered SNPs/loci or at the very least SNPs unique to other populations that are not of Caucasian ancestry. For example, one meta-analysis on Oriental populations found five Oriental-specific loci near *CAPZA1*, *FIGN*, *ENPEP*, *NPR3*, and *PTPN11* (near *C12orf51*) associated with hypertension [22]. Either the differences in environmental exposures/lifestyle factors or genetic background can explain why ethnic/racial susceptibility loci exist. Nevertheless, as even a small increase in blood pressure can increase the risk of cardiovascular diseases, the biological pathways identified by these SNPs would still be useful in resolving many of the open questions regarding blood pressure pathophysiology.

## 3. Biological Pathways Involved with Blood Pressure Pathophysiology

Mendelian forms of hypertension and germline mutations causing early-onset hypertension have highlighted biological pathways that involve renal salt handling (*WNK1*, *WNK4*, *KLHL3*, and *CUL3*), ion transport (*CACNA1D*, *CACNA1H*, *KCNJ5*, *SCNN1B*, and *SCNN1G*), corticosteroidogenesis (*CYP11B2*, *HSD11B2*, *NR3C2*, *CYP11B1*, and *CYP17A1*), and vascular tone (*PDE3A*) to regulate blood [41–44]. Thus, not surprisingly, GWASs have identified SNPs in or near to genes involved with these biological pathways associated with primary hypertension. In fact, one of the first few high-throughput genotyping was performed on only genes underlying monogenic hypertension and hypotension (not genome-wide) which found two renal sodium regulatory genes (*KCNJ1* and *NR3C2*) to have SNPs associated with blood pressure in the general population [45].

### 3.1. Renal Salt Handling

One interesting SNP putatively involving renal salt-handling pathway was only linked to hypertension in an extreme case-control GWAS design [25]. This SNP, rs13333226, on chromosome 16 is in the 5′ region of *UMOD* (combined *P* value of 3.6 × 10^−11^). The minor G allele of this SNP had an OR of 0.87 (95% CI: 0.84–0.91) for hypertension, with the subject having the minor G allele having decreased urinary uromodulin and better renal function. The exclusive expression of uromodulin, the protein encoded by *UMOD*, in the thick portion of the ascending limb of Henle suggests that the SNP exerts its effect through sodium homeostasis [25]. Also based on renal expression, SNPs in or near to *PAPPA2* and *ADAMTS7* (rs61823001 and rs62011052, resp., [8]) are expected to play a role in the renal salt-handling pathway. Interestingly in regard to the protein encoded by *ADAMTS7*, angiotensin II stimulation induced renal expression of the protein [46]. Similarly, renal cortex expression of *PAPPA2* in Dahl salt-sensitive rats responded to changes of salt diet supporting a role of the SNP in the renal salt-handing pathway [47]. SNPs in *FAM186B* and *ARHGAP24* on the other hand are postulated to play a role in renal function based on involvement with kidney diseases. Combining whole exome sequencing and homozygosity mapping in consanguineous families, *FAM186B* was identified as a novel candidate gene for monogenic, recessive nephronophthisis-related ciliopathies [48]. *ARHGAP24* on the other hand is thought to play a role in renal cell carcinoma and focal segmental glomerulosclerosis most likely through RhoA and Rac1 signaling pathways [49, 50].

### 3.2. Ion Transport

Several SNPs in genes involved with ion transport have been associated with blood pressure (e.g., *ATP2B1*, *CACNA1D*, *CACNA2D2*, *CACNB2*, *KCNK3*, *SLC4A7*, and *SLC39A8*; Table 1). Of these, the one most studied and replicated are SNPs in *ATP2B1* [9, 18, 22, 51]. Confirming the role of *ATP2B1* in blood pressure regulation is the vascular smooth muscle cell-specific knockout of *ATP2B1* mice which had higher systolic blood pressure and significantly increased phenylephrine-induced vasoconstrictions [52]. Similarly, silencing of *ATP2B1* through injection of an SiRNA complex into mouse tail veins led to an increase in blood pressure and an increase in contractile response to phenylephrine [53]. These results support that *ATP2B1* genetic variants are the causative gene for the association with blood pressure seen in GWASs. The other gene encoding an ion channel with significant supporting evidence is *CACNA1D*. This is because gain of function mutations in *CACNA1D* have been found to be causal for primary aldosteronism and for aldosterone-producing cell clusters [42, 54, 55]. As aldosterone is a key regulator of blood pressure, even small changes which may not pass the clinical threshold for primary aldosteronism may be causal for increase in blood pressure. Elevation of aldosterone may also be the mechanism of action for the other ion channels associated with primary hypertension as mutations in the ATPase Na^+^/K^+^ transporting subunit alpha 1 and G protein-activated inward rectifier K^+^ channel 4 have also been found causal for primary aldosteronism and aldosterone-producing cell clusters [55, 56].

### 3.3. Corticosteroidogenesis

Surprisingly in the sense that corticosteroids can highly affect blood pressure, only 2 cytochrome P450 enzyme genes involved with corticosteroidogenesis have been linked to hypertension by GWASs—*CYP17A1* and *CYP21A2*. And of that, only SNPs in the *CYP17A1* gene have been replicated, though even then with inconsistent results. *CYP17A1* encodes 17*α*-hydroxylase which is essential to the synthesis of cortisol precursors. Therefore, alteration of this gene can cause a deficiency in 17*α*-hydroxylase and thus cortisol, which affects blood pressure [57]. Supporting the role of *CYP17A1* in blood pressure regulation is the SNP rs11191548, a SNP near the *CYP17A1* gene that has been consistently associated with blood pressure in both East Asian cohorts and Caucasian cohorts [10, 17, 18, 58–60]. Patients harboring the risky C allele had lower PRA and K+ levels similar to patients with 17*α*-hydroxylase deficiency, suggesting that the SNP (which is actually in the noncoding region of the gene *CNNM2*) has an effect on the enzymatic activity of *CYP17A1* [58]. One hypothesis as to why inconsistent results occur with GWAS is if the association found between the lead SNP is indirect whereby the signal produced is actually caused by a synthetically linked rarer variant in linkage disequilibrium with the identified tag SNP. This could be the case with the lead SNP rs1004467 which was identified from the CHARGE + Global BPgen meta-analysis [9]. In an Oriental cohort (from Korea), rs1004467 was found to have a modest association with hypertension in prediabetic subjects and a significant association with augmentation index in diabetic subjects [61]. However, in another Oriental cohort with similar ethnic background (from China), rs1004467 association with hypertension/blood pressure was not found in children [62]. As such, perhaps the causal SNP is not rs1004467 as identified by the initial GWAS meta-analysis but a tag SNP with poor penetrance. Interestingly, rs1004467 is in linkage disequilibrium with rs138009835, a functional SNP located 1800 bases upstream of the transcription site of *CYP17A1*. In vitro gene reporter gene assays and clinical functional experiments found the minor alleles to have reduced mRNA expression of *CYP17A1* and reduced aldosterone excretion [63]. To note, both rs1004467 and rs11191548 are associated with a reduction in both visceral and subcutaneous fat mass in Japanese women [64].

### 3.4. Vascular Tone

Interestingly, although only one of the fifteen monogenic hypertension genes is postulated to mediate an effect through the vasculature, SNPs associated with blood pressure and primary hypertension are enriched in genes that are expressing their proteins in vascular smooth muscle and endothelial cells [11, 12, 65–67]. This is consistent with vascular tone playing a primary role in blood pressure regulation. Many of these genes, however, may have been reported as the causal genes due to their proximity to the SNP in question and their likelihood of playing a role in blood pressure regulation rather than due to real functional data [68]. For example, the reported gene for rs7129220, a SNP downstream to the *ADM* gene in the noncoding RNA *CAND1.11* gene, was the *ADM* gene as adrenomedullin the protein encoded by *ADM* plays a role in vasodilation [69]. Oppositely, the reported genes for rs633185 are *FLJ32810*-*TMEM133*, even though the SNP is within the intron of *ARHGAP42* (Table 1). As a candidate gene for blood pressure regulation, *ARHGAP42* has many functional evidence to be the causal gene as reduced expression of *ARHGAP42* in mice elevated blood pressure [70]. To note, rs633185 is in high linkage disequilibrium with rs604723, another SNP in the intron of *ARHGAP42*, and the minor T allele is a functional variant that increases *ARHGAP42* expression by promoting serum response factor binding to a smooth muscle-selective regulatory element [71]. Based on this strong functional data, rs604723 is most likely the causative SNP at this locus. rs6271 in exon 11 of the *DBH* gene on the other hand is one of the rare times where GWASs had managed to directly identify a missense variant which is probably damaging to the protein dopamine *β*-hydroxylase according to PolyPhen-2 prediction [72]. Concurringly, severe orthostatic syndrome (postural hypotension) were found to be caused by truncating, splice site, or missense mutations in the *DBH* gene [73].

## 4. Conclusion

Although some of the SNPs identified by GWAS on primary hypertension associates with similar biological pathways as Mendelian or early-onset forms of hypertension (validating the study approach), none of the SNPs identified had a large size effect (≤1 mmHg) to be of significance to an individual patient. The ultimate goals of performing these GWASs are to determine the genetic factors regulating blood pressure that can be used to make predictions about who is at risk of developing hypertension and to identify the biological pathways of the disease allowing for identification of novel targets for treatment or even prevention strategies. As currently no direct clinical application of these GWAS findings can be made, it is still debatable whether GWAS is the best approach to identify the biological underpinnings of primary hypertension. Even though yet-to-be-discovered Oriental-specific loci or rare SNPs that might have larger effect size may increase the variance for blood pressure that can be explained by genetic variation, information on epigenetic modulation (e.g., DNA methylation, posttranslational modifications of proteins, or even gut microbiota [20, 74–78]) may still be needed to explain the total heritability of raised blood pressure which cannot be captured by GWASs.

## Figures and Tables

**Table 1:** Loci associated with blood pressure and/or hypertension that have been identified through large-scale studies in the past decade.

Locus name	SNP	Chr ID	Chr position	Coded allele	Best trait	Effect size of best trait (OR beta)	Coded allele frequency	Reporting article
*2q36.3*	rs2972146	2	226,235,982	T	DBP	0.17	0.19	Surendran et al. [29]
*7q32.1*	rs4728142	7	128,933,913	A	SBP	−0.224	0.29	Surendran et al. [29]
*ABHD17C*	rs35199222	15	80,720,696	A	SBP	0.322	0.18	Hoffmann et al. [8], Warren et al. [26]
*ABHD17C*	rs11634851	15	80,736,624	G	SBP	0.316	0.461	Wain et al. [27]
*ABLIM3-SH3TC2*	rs9687065	5	149,011,577	A	DBP	0.26	0.16	Kato et al. [20]
*ACE*	rs4308	17	63,482,264	A	DBP	0.213	0.24	Hoffmann et al. [8], Warren et al. [26]
*ACOX1*	rs2467099	17	75,952,964	T	SBP	−0.307	0.18	Hoffmann et al. [8], Warren et al. [26]
*ADAMTS7-MORF4L1*	rs62012628	15	78,777,658	T	DBP	−0.238	0.34	Hoffmann et al. [8], Warren et al. [26]
*ADAMTS7-MORF4L1*	rs62011052	15	79,156,983	C	PP	−0.28	0.14	Hoffmann et al. [8]
*ADAMTS8*	rs11222084	11	130,403,335	T	PP	0.337	0.21	Wain et al. [19]
*ADAMTS9*	rs918466	3	64,724,577	A	DBP	−0.204	0.35	Ehret et al. [12]
*ADCY3*	rs55701159	2	24,916,727	T	DBP	0.285	0.1	Warren et al. [26]
*ADM*	rs360157	11	9,732,674	T	SBP	0.413	0.44	Ehret et al. [12]
*ADM*	rs7129220	11	10,350,538	A	SBP	−0.619	0.058	Ehret et al. [18]
*ADM*	rs7129220	11	10,350,538	A	DBP	−0.299	0.058	Ehret et al. [18]
*ADO*	rs10995311	10	62,805,174	G	DPB	−0.20	0.38	Liu et al. [23], Surendran et al. [29]
*ADRB1*	rs2782980	10	114,021,768	T	PP	−0.338	0.28	Wain et al. [19]
*ADRB1-RNU6-709P*	rs10787517	10	114,055,047	A	SBP	0.442	0.616	Wain et al. [27]
*AGT*	rs2004776	1	230,712,956	T	SBP	0.42	0.41	Johnson et al. [30]
*AKT2*	rs9710247	19	40,254,542	G	DBP	0.252	0.44	Wain et al. [27]
*AMH-SF3A2*	rs740406	19	2,232,222	A	PP	−0.55	0.21	Kato et al. [20]
*ARHGAP12*	rs10826995	10	31,793,730	T	PP	−0.212	0.3	Hoffmann et al. [8], Warren et al. [26]
*ARHGAP24*	rs2014912	4	85,794,517	T	SBP	0.62	0.19	Kato et al. [20]
*ARNTL*	rs900145	11	13,272,358	G	DBP	−0.25	0.43	Liu et al. [23]
*ARVCF*	rs12628032	22	19,980,457	T	PP	0.24	0.27	Hoffmann et al. [8], Warren et al. [26]
*ARVCF*	rs4819852	22	20,000,644	A	PP	0.261	0.29	Wain et al. [27]
*ATP2B1*	rs2681472	12	89,615,182	A	DBP	0.5	0.83	Levy et al. [9]
*ATP2B1*	rs2681492	12	89,619,312	T	SBP	1.26	0.21	Levy et al. [9]
*ATP2B1*	rs17249754	12	89,666,809	A	BP	0.8	0.35	Kelly et al. [31]
*BAT2-BAT5*	rs805303	6	31,648,589	G	SBP	0.376	0.44	Johnson et al. [30]
*BDNF*	rs11030119	11	27,706,555	A	DBP	−0.163	0.26	Hoffmann et al. [8], Warren et al. [26]
*BLK-GATA4*	rs2898290	8	11,576,400	C	SBP	NA	0.38	Ho et al. [33]
*C10orf107*	rs4590817	10	61,707,795	C	DBP	0.436	0.16	Wain et al. [27]
*C10orf107*	rs1530440	10	61,764,833	T	DBP	0.19	0.15	Newton-Cheh et al. [10]
*C10orf32, C10orf32-ASMT*	rs4409766	10	102,856,906	T	SBP	1.24	0.71	Lu et al. [51]
*C17orf82-TBX2*	rs2240736	17	61,408,032	T	MAP	0.35	0.35	Kato et al. [20]
*C20orf187*	rs1887320	20	10,985,350	A	SBP	0.78	0.53	Lu et al. [51]
*C2orf43*	rs2289081	2	20,682,080	C	PP	−0.223	0.31	Hoffmann et al. [8], Warren et al. [26]
*C5orf56*	rs2188962	5	132,435,113	T	DBP	−0.2	0.14	Liu et al. [23], Surendran et al. [29]
*CACNA1D*	rs9810888	3	53,601,568	G	DBP	0.39	0.39	Lu et al. [51]
*CACNA2D2*	rs743757	3	50,438,947	C	DBP	0.245	0.36	Hoffmann et al. [8], Warren et al. [26]
*CACNB2*	rs1813353	10	18,418,519	C	DBP	0.332	0.34	Wain et al. [27]
*CACNB2*	rs11014166	10	18,419,869	A	DBP	0.46	0.21	Levy et al. [9]
*CAMKV-ACTBP13*	rs36022378	3	49,876,272	T	DBP	−0.202	0.11	Hoffmann et al. [8], Warren et al. [26]
*CAPZA1*	rs10745332	1	112,646,431	A	SBP	0.96	0.82	Lu et al. [51]
*CASC15*	rs6911827	6	22,130,372	T	SBP	0.296	0.30	Hoffmann et al. [8], Warren et al. [26]
*CASZ1*	rs880315	1	10,736,809	T	SBP	−0.475	0.39	Ehret et al. [12]
*CCDC141*	rs79146658	2	178,921,341	T	DBP	−0.311	0.03	Hoffmann et al. [8], Warren et al. [26]
*CCDC41-CEP83-RN7SL483P*	rs139236208	12	94,486,966	A	PP	−0.363	0.04	Hoffmann et al. [8], Warren et al. [26]
*CCNE1*	rs62104477	19	29,804,084	T	DBP	0.177	0.19	Hoffmann et al. [8], Warren et al. [26]
*CD34*	rs12731740	1	207,851,475	T	PP	−0.249	0.08	Warren et al. [26]
*CDC42BPA*	rs10916082	1	227,064,925	A	DBP	−0.177	0.27	Warren et al. [26]
*CDH13*	rs7500448	16	83,012,185	A	PP	0.329	0.17	Hoffmann et al. [8], Warren et al. [26]
*CDH17*	rs2446849	8	94,091,269	T	SBP	−0.63	0.22	Zhu et al. [32]
*CELA2A*	rs1042010	1	15,467,418	A	SBP	0.412	0.19	Hoffmann et al. [8], Warren et al. [26]
*CELA2A*	rs3820068	1	15,471,702	A	SBP	0.425	0.19	Wain et al. [27]
*CEP164*	rs8258	11	117,412,960	T	PP	0.236	0.47	Hoffmann et al. [8], Warren et al. [26]
*CEP68*	rs74181299	2	65,056,838	T	PP	0.23	0.46	Hoffmann et al. [8], Warren et al. [26]
*CERS5*	rs7302981	12	50,144,032	A	DBP	0.249	0.30	Liu et al. [23], Surendran et al. [29]
*CFDP1*	rs11643209	16	75,297,146	T	SBP	−0.339	0.47	Hoffmann et al. [8], Warren et al. [26]
*CHIC2*	rs871606	4	53,933,078	T	PP	0.429	0.21	Wain et al. [19]
*chr15mb95*	rs12906962	15	94,768,842	T	DBP	−0.221	0.42	Hoffmann et al. [8], Warren et al. [26]
*chr1mb25*	rs6686889	1	24,703,979	T	DBP	0.185	0.37	Warren et al. [26]
*chr1mb9*	rs9662255	1	9,381,890	A	PP	−0.207	0.41	Hoffmann et al. [8], Warren et al. [26]
*CHST12-LFNG*	rs2969070	7	2,472,910	A	DBP	−0.205	0.21	Ehret et al. [12]
*CMIP*	rs8059962	16	81,540,592	T	DBP	−0.170	0.45	Warren et al. [26]
*CNNM2*	rs11191548	10	103,086,421	C	SBP	1.082	0.09	Wain et al. [27]
*COL21A1*	rs1925153	6	56,237,982	T	PP	−0.21	0.44	Liu et al. [23]
*CPEB4*	rs72812846	5	173,950,633	A	DBP	−0.209	0.11	Hoffmann et al. [8], Warren et al. [26]
*CRACR2B*	rs7126805	11	828,916	A	PP	0.222	<0.01	Warren et al. [26]
*CRK*	rs12941318	17	1,430,304	T	SBP	−0.269	0.37	Hoffmann et al. [8], Warren et al. [26]
*CRYAA-SIK1-RRP1B*	rs12627651	21	43,340,723	A	SBP	0.503	0.19	Ehret et al. [12], Surendran et al. [29]
*CSK*	rs1378942	15	74,785,026	A	DBP	0.371	0.65	Wain et al. [27]
*CYB561-LOC342541*	rs4459609	17	63,471,587	A	DBP	0.198	0.61	Wain et al. [27]
*CYP17A1-NT5C2*	rs1004467	10	102,834,750	A	SBP	1.2	0.16	Levy et al. [9], Newton-Cheh et al. [10]
*CYP1A1-ULK3*	rs6495122	15	74,833,304	A	DBP	0.45	0.29	Levy et al. [9], Newton-Cheh et al. [10]
*CYP2C19*	rs4494250	10	94,804,000	A	DPB	0.21	0.22	Liu et al. [23]
*DBH*	rs6271	9	133,657,152	T	DBP	−0.423	0.04	Ehret et al. [12]
*DNM3*	rs12405515	1	172,388,301	T	DBP	−0.165	0.47	Hoffmann et al. [8], Warren et al. [26]
*DPEP1*	rs1126464	16	89,637,957	C	DBP	0.275	0.26	Liu et al. [23], Surendran et al. [29]
*EBF1*	rs11953630	5	158,418,394	T	DBP	−0.281	0.18	Johnson et al. [30]
*EBF2*	rs6557876	8	26,043,159	T	SBP	−0.411	0.33	Wain et al. [27]
*ENPEP*	rs6825911	4	110,460,482	C	DBP	0.39	0.42	Kato et al. [22]
*ESR1*	rs13192976	6	151,991,280	A	PP	−0.332	0.21	Hoffmann et al. [8], Warren et al. [26]
*FAF1*	rs147696085	1	50,556,195	G	PP	0.298	0.06	Hoffmann et al. [8]
*FAM186B*	rs7977389	12	49,587,939	T	PP	0.237	0.18	Hoffmann et al. [8]
*FAM208A*	rs9827472	3	56,692,618	T	DBP	−0.177	0.46	Hoffmann et al. [8], Warren et al. [26]
*FBLN5*	rs2244643	14	91,892,678	A	PP	−0.213	0.29	Hoffmann et al. [8]
*FBN2*	rs6595838	5	128,532,506	A	SBP	0.344	0.41	Hoffmann et al. [8], Warren et al. [26]
*FBXL19*	rs72799341	16	30,925,422	A	DBP	0.185	0.27	Hoffmann et al. [8], Warren et al. [26]
*FER1L5*	rs7599598	2	96,686,103	A	DBP	−0.31	0.42	Ganesh et al. [34]
*FERMT2*	rs9888615	14	52,910,822	T	SBP	−0.318	0.36	Hoffmann et al. [8], Warren et al. [26]
*FGD5*	rs11128722	3	14,916,619	A	SBP	−0.383	0.41	Ehret et al. [12]
*FGF5*	rs16998073	4	80,263,187	T	DBP	0.21	0.23	Newton-Cheh et al. [10]
*FGGY-HSD52*	rs3889199	1	59,188,070	A	PP	0.351	0.14	Hoffmann et al. [8], Warren et al. [26]
*FIGN-PRPS1P1*	rs16849211	2	164,043,173	T	PP	0.364	0.23	Wain et al. [27]
*FIGN-PRPS1P1*	rs1446468	2	164,106,976	C	SBP	0.538	0.55	Wain et al. [27]
*FIGN-GRB14*	rs16849225	2	164,050,310	C	SBP	0.75	0.23	Ehret et al. [18], Kato et al. [22], Wain et al. [19]
*FLJ32810-TMEM133*	rs633185	11	100,722,807	G	SBP	−0.565	0.36	Johnson et al. [30]
*FN1*	rs1250259	2	215,435,759	A	PP	−0.314	0.23	Hoffmann et al. [8], Warren et al. [26]
*FNDC1*	rs449789	6	159,278,093	C	PP	0.359	0.15	Hoffmann et al. [8], Warren et al. [26]
*FOSL2*	rs7562	2	28,412,873	T	SBP	0.263	0.50	Warren et al. [26]
*FRMD3*	rs115795127	9	83,378,986	T	BP	NA	NR	Liang et al. [35]
*FURIN-FES*	rs2521501	15	90,894,158	T	SBP	0.65	0.21	Johnson et al. [30]
*GATA2*	rs62270945	3	128,483,046	T	PP	0.607	0.01	Hoffmann et al. [8], Warren et al. [26]
*GJA1*	rs11154027	6	121,460,244	T	PP	0.207	0.38	Warren et al. [26]
*GNAS-EDN3*	rs6015450	20	59,176,062	G	SBP	0.896	0.10	Johnson et al. [30]
*GOSR2*	rs17608766	17	46,935,905	T	SBP	−0.556	0.05	Johnson et al. [30]
*GPAT2-FAHD2CP*	rs2579519	2	96,009,418	T	DBP	−0.197	0.41	Warren et al. [26]
*GPATCH2*	rs12408022	1	217,545,447	T	DBP	0.198	0.26	Hoffmann et al. [8], Warren et al. [26]
*GPR20*	rs34591516	8	141,356,987	T	SBP	0.323	0.05	Surendran et al. [29]
*GPR20*	rs78192203	8	141,364,973	T	BP	NA	NR	Liang et al. [35]
*GPR98/ARRDC3*	rs10474346	5	91,268,322	C	DBP	1.1	0.31	Fox et al. [36]
*GTF2B*	rs10922502	1	88,894,475	A	SBP	−0.382	0.34	Hoffmann et al. [8], Warren et al. [26]
*GUCY1A3*	rs13143871	4	155,698,052	T	SBP	0.96	0.80	Lu et al. [51]
*GUCY1A3-GUCY1B3*	rs13139571	4	155,724,361	C	DBP	0.26	0.21	Johnson et al. [30]
*GYPA_HHIP*	rs4292285	4	144,350,802	T	DBP	0.177	0.41	Hoffmann et al. [8]
*HAAO-RNU6-242P-AC016735.1*	rs13403122	2	42,851,618	C	DBP	0.226	0.20	Hoffmann et al. [8], Warren et al. [26]
*HDAC9*	rs2107595	7	19,009,765	A	PP	0.31	0.25	Kato et al. [20]
*HFE*	rs1799945	6	26,090,951	G	DBP	0.457	0.09	Johnson et al. [30]
*HFE*	rs1800562	6	26,092,913	A	DBP	0.394	0.06	Wain et al. [27]
*HIPK2*	rs1011018	7	139,763,465	A	SBP	−0.329	0.35	Warren et al. [26]
*HIVEP3*	rs7515635	1	41,942,399	T	SBP	0.336	0.47	Ehret et al. [12]
*HM13-ID1*	rs6060114	20	31,581,870	T	DBP	0.267	0.27	Hoffmann et al. [8]
*HNF4G-RNU2-54P*	rs1449544	8	75,679,645	A	PP	0.183	0.41	Hoffmann et al. [8]
*HOTTIP*	rs1859168	7	27,202,740	C	DBP	0.436	0.92	Wain et al. [27]
*HOXA3*	rs6969780	7	27,119,517	C	BP	NA	NR	Liang et al. [35]
*HOXA-EVX1*	rs17428471	7	27,298,248	T	SBP	1.2	0.08	Franceschini et al. [24]
*HOXB7*	rs7406910	17	48,610,894	T	SBP	−0.456	0.12	Surendran et al. [29]
*HRCT1*	rs76452347	9	35,906,474	T	DBP	−0.25	0.15	Liu et al. [23]
*HSD52-LOC105378756*	rs10889130	1	59,148,708	A	PP	0.288	0.33	Wain et al. [27]
*HSPB7*	rs1048238	1	16,015,154	T	SBP	0.366	0.02	Wain et al. [27]
*IGFBP3*	rs11977526	7	45,968,511	A	DBP	0.3	0.44	Zhu et al. [32], Liu et al. [23]
*INPP5B*	rs871524	1	37,945,773	A	PP	0.228	0.33	Wain et al. [27]
*INSR*	rs7248104	19	7,224,420	A	PP	−0.20	0.35	Liu et al. [23]
*INSR*	rs36047283	19	7,255,690	G	SBP	0.801	0.11	Wain et al. [27]
*ITGA11*	rs1563894	15	68,343,437	A	SBP	−0.093	0.18	Parmar et al. [37]
*JAG1*	rs1327235	20	10,988,382	G	DBP	0.302	0.46	Johnson et al. [30]
*JAG1-LOC101929395*	rs6040076	20	10,678,234	C	PP	0.285	0.49	Wain et al. [27]
*KCNH4-HSD17B1*	rs79089478	17	42,165,223	T	PP	0.584	0.01	Warren et al. [26]
*KCNK3*	rs1275988	2	26,691,496	T	SBP	−0.6	0.41	Ganesh et al. [34]
*KIAA0753*	rs7226020	17	6,570,508	T	PP	−0.256	0.38	Hoffmann et al. [8], Warren et al. [26]
*KIAA1462*	rs9337951	10	30,028,144	A	PP	0.28	0.26	Hoffmann et al. [8], Warren et al. [26]
*L3MBTL4*	rs403814	18	6,282,594	A	BP	1.15	NR	Liu et al. [23]
*LHFPL2*	rs10057188	5	78,541,966	A	PP	−0.205	0.24	Hoffmann et al. [8], Warren et al. [26]
*LINC01615-THBS2*	rs1322639	6	169,187,008	A	PP	0.316	0.33	Hoffmann et al. [8], Warren et al. [26]
*LMO1*	rs110419	11	8,231,306	A	DBP	0.159	0.43	Surendran et al. [29]
*LOC101928278*	rs10932679	2	216,787,868	T	PP	0.226	0.19	Wain et al. [27]
*LOC102723446*	rs10260816	7	45,970,501	G	PP	0.298	0.43	Wain et al. [27]
*LOC105369687-LOC105369688*	rs73075659	12	20,220,607	G	SBP	0.357	0.31	Wain et al. [27]
*LOC105370003*	rs11067763	12	115,760,536	A	DBP	0.51	0.62	Lu et al. [51]
*LOC105371811-LOC105371812*	rs79917357	17	48,747,312	A	SBP	0.342	0.17	Wain et al. [27]
*LOC105374567-LOC102723854*	rs72876037	2	42,967,456	T	SBP	0.534	0.12	Wain et al. [27]
*LOC105379231*	rs9693857	8	9,409,607	T	SBP	0.337	0.45	Wain et al. [27]
*LOC107986913-LOC105379224*	rs7826238	8	8,529,585	T	SBP	0.335	0.47	Wain et al. [27]
*LOC283335*	rs73099903	12	53,046,995	T	SBP	0.768	0.06	Wain et al. [27]
*LRP12/ZFPM2*	rs35783704	8	104,954,030	A	SBP	−0.609	0.03	Wain et al. [27]
*LRRC10B-SYT7*	rs751984	11	61,510,774	T	MAP	0.33	0.27	Kato et al. [20], Ehret et al. [12]
*LSP1-TNNT3*	rs661348	11	1,884,062	T	MAP	−0.65	0.42	Johnson et al. [30]
*MAP4*	rs319690	3	47,885,994	T	DBP	0.282	0.41	Wain et al. [19]
*MAPK4-MRO*	rs36010659	18	50,757,579	T	PP	0.25	0.12	Hoffmann et al. [8], Warren et al. [26]
*MCF2L*	rs9549328	13	112,981,842	T	SBP	0.318	0.22	Hoffmann et al. [8], Warren et al. [26]
*MECOM*	rs419076	3	169,383,098	T	SBP	0.409	0.42	Johnson et al. [30]
*METTL21A-AC079767.3*	rs55780018	2	207,661,416	T	SBP	−0.391	0.35	Hoffmann et al. [8], Warren et al. [26]
*MIR1263*	rs16833934	3	164,019,462	G	DBP	−1.63	0.31	Simino et al. [38]
*MKLN1*	rs13238550	7	131,374,297	A	SBP	0.331	0.33	Warren et al. [26]
*MOV10*	rs12129649	1	112,688,881	T	DBP	0.548	0.06	Wain et al. [27]
*MRAS*	rs2306374	3	138,401,110	T	DBP	−0.184	0.08	Hoffmann et al. [8], Warren et al. [26]
*MRC2*	rs740698	17	62,689,790	T	PP	−0.228	0.41	Warren et al. [26]
*MSRA*	rs11249992	8	10,362,902	A	SBP	0.293	0.38	Wain et al. [27]
*MTAP*	rs4364717	9	21,801,531	A	DBP	−0.175	0.43	Warren et al. [26]
*MTF1-SF3A3*	rs4360494	1	37,990,219	C	PP	0.278	0.38	Hoffmann et al. [8], Warren et al. [26]
*MTHFR*	rs17367504	1	11,802,721	G	DBP	0.526	0.15	Wain et al. [27]
*MTHFR-NPPB*	rs4846049	1	11,790,308	T	DBP	−0.55	0.37	Johnson et al. [30]
*MYEOV*	rs67330701	11	69,312,240	T	DBP	−0.367	0.12	Hoffmann et al. [8], Warren et al. [26]
*MYH6*	rs452036	14	23,396,676	A	PP	−0.282	0.34	Liu et al. [23], Surendran et al. [29]
*NADK-CPSF3L*	rs139385870	1	1,754,504	D	SBP	−0.352	0.33	Hoffmann et al. [8], Warren et al. [26]
*NFKBIA*	rs8904	14	35,402,011	A	SBP	0.377	0.40	Wain et al. [27]
*NME7*	rs7519279	1	169,238,123	G	PP	0.218	0.13	Hoffmann et al. [8]
*NOS3*	rs3918226	7	150,993,088	T	DBP	0.83	0.03	Johnson et al. [30]
*NOTCH3*	rs10418305	19	15,167,997	C	PP	−0.282	0.13	Hoffmann et al. [8]
*NOV*	rs2071518	8	119,423,572	T	PP	0.312	0.32	Wain et al. [19]
*NOX4*	rs2289125	11	89,491,285	A	PP	−0.377	0.32	Hoffmann et al. [8], Warren et al. [26]
*NPNT*	rs13112725	4	105,990,585	C	SBP	0.435	0.34	Hoffmann et al. [8], Warren et al. [26]
*NPPA-AS1, NPPA*	rs12744757	1	11,846,764	T	SBP	0.695	0.06	Wain et al. [27]
*NPR1*	rs35479618	1	153,689,947	A	SBP	1.34	0.01	Liu et al. [23]
*NPR3-C5orf23*	rs1173771	5	32,814,922	C	SBP	0.63	0.34	Johnson et al. [30], Kato et al. [22]
*OBFC1*	rs4387287	10	103,918,139	A	SBP	0.338	0.32	Surendran et al. [29]
*OR5B12*	rs11229457	11	58,439,730	T	SBP	−0.312	0.22	Surendran et al. [29]
*OSR1*	rs1344653	2	19,531,084	A	PP	−0.27	0.38	Kato et al. [20]
*PABPC4*	rs4660293	1	39,562,508	G	DBP	0.27	0.10	Liu et al. [23]
*PALLD-chr4mb174*	rs1566497	4	168,795,997	A	PP	0.236	0.23	Hoffmann et al. [8], Warren et al. [26]
*PAPPA2*	rs61823001	1	176,664,440	G	PP	0.31	0.03	Hoffmann et al. [8]
*PAX2*	rs112184198	10	100,844,757	A	SBP	−0.659	0.05	Hoffmann et al. [8], Warren et al. [26]
*PDE10A*	rs147212971	6	165,764,963	T	DBP	−0.360	0.13	Hoffmann et al. [8], Warren et al. [26]
*PDE3A*	rs12579720	12	20,020,830	C	DBP	−0.32	0.46	Kato et al. [20]
*PDE5A*	rs66887589	4	119,588,124	T	DBP	−0.215	0.50	Hoffmann et al. [8], Warren et al. [26]
*PHACTR1*	rs9349379	6	12,903,725	A	SBP	0.289	0.38	Surendran et al. [29]
*PHIP*	rs10943605	6	78,945,760	A	DBP	0.18	0.49	Liu et al. [23]
*PIK3CG*	rs17477177	7	106,771,412	T	PP	−0.418	0.17	Wain et al. [19]
*PKHD1*	rs13205180	6	51,967,696	T	DBP	0.168	0.34	Hoffmann et al. [8], Warren et al. [26]
*PKN2-AS1*	rs61767086	1	88,600,899	G	PP	0.413	0.14	Wain et al. [27]
*PLCB1*	rs6108168	20	8,645,624	A	DBP	−0.211	0.38	Warren et al. [26]
*PLCD3*	rs12946454	17	45,130,754	T	SBP	0.28	0.21	Newton-Cheh et al. [10]
*PLCE1*	rs932764	10	94,136,183	G	SBP	0.484	0.43	Johnson et al. [30]
*PLCE1*	rs932764	10	94,136,183	G	SBP	0.484	0.44	Ehret et al. [18]
*PLEKHA7*	rs177542	11	16,901,107	A	DBP	0.243	0.50	Wain et al. [27]
*PLEKHA7-NUCB2*	rs381815	11	16,880,721	T	SBP	0.84	0.21	Levy et al. [9]
*PLEKHG1*	rs17080102	6	150,683,634	C	DBP	−0.74	0.12	Franceschini et al. [24]
*PNPT1*	rs1975487	2	55,581,918	A	DBP	−0.217	0.32	Ehret et al. [12]
*POC5-SV2C*	rs10078021	5	75,742,606	T	DBP	−0.164	0.46	Hoffmann et al. [8], Warren et al. [26]
*PPL*	rs12921187	16	4,893,018	T	DBP	−0.174	0.41	Hoffmann et al. [8],Warren et al. [26]
*PPP2R5E*	rs8016306	14	63,461,828	A	SBP	0.335	0.41	Warren et al. [26]
*PRDM11*	rs11442819	11	45,186,590	I	PP	−0.279	0.13	Hoffmann et al. [8], Warren et al. [26]
*PRDM16*	rs2493292	1	3,412,095	T	SBP	0.42	0.13	Liu et al. [23]
*PRDM6-SUMO1P5*	rs337100	5	123,210,816	A	PP	0.277	0.40	Wain et al. [27]
*PRDM6-CSNK1G3*	rs13359291	5	123,140,763	A	SBP	0.53	0.28	Kato et al. [20]
*PRDM8-FGF5*	rs1902859	4	80,236,549	C	SBP	1.34	0.41	Lu et al. [51]
*PRDM8-FGF5*	rs1458038	4	80,243,569	T	DBP	0.403	0.30	Wain et al. [27]
*PREX1*	rs6095241	20	48,692,260	A	DBP	−0.168	0.46	Surendran et al. [29]
*PRKAG1*	rs1126930	12	49,005,349	C	PP	0.5	0.02	Surendran et al. [29]
*PRKCE*	rs11690961	2	46,136,197	A	PP	0.34	0.04	Hoffmann et al. [8], Warren et al. [26]
*PRKD3*	rs13420463	2	37,290,423	A	SBP	0.356	0.49	Hoffmann et al. [8], Warren et al. [26]
*PROCR*	rs867186	20	35,176,751	A	DBP	0.265	0.11	Surendran et al. [29]
*PRRC2A-BAG6*	rs151168737	6	31,638,615	A	DBP	0.249	0.46	Wain et al. [27]
*PSMD5*	rs10760117	9	120,824,459	T	SBP	0.334	0.42	Ehret et al. [12], Liu et al. [23]
*PYY*	rs62080325	17	43,983,263	A	PP	−0.186	0.21	Warren et al. [26]
*RABGAP1*	rs10818775	9	122,993,292	C	PP	0.254	0.30	Hoffmann et al. [8]
*RAPSN, PSMC3, SLC39A13*	rs7103648	11	47,440,232	A	DBP	−0.203	0.33	Ehret et al. [12]
*RBM47*	rs35529250	4	40,426,074	T	SBP	−1.537	<0.01	Surendran et al. [29]
*RCOR2*	rs4980532	11	63,913,247	T	PP	0.301	0.56	Wain et al. [27]
*RGL3*	rs167479	19	11,416,089	T	DBP	−0.33	0.49	Liu et al. [23], Surendran et al. [29]
*RNF207*	rs709209	1	6,218,354	A	PP	0.199	0.36	Surendran et al. [29]
*RP11-273G15.2*	rs62524579	8	142,979,538	A	DBP	−0.175	0.48	Hoffmann et al. [8], Warren et al. [26]
*RP11-321F6.1*	rs7178615	15	66,576,734	A	DBP	−0.179	0.36	Warren et al. [26]
*RP11-435J9.2-TLN2*	rs956006	15	62,516,340	C	PP	0.188	0.23	Hoffmann et al. [8]
*RP11-439C8.2*	rs143112823	3	154,990,178	A	DBP	−0.403	0.06	Hoffmann et al. [8], Warren et al. [26]
*RP11-61O1.1*	rs9323988	14	98,121,293	T	PP	−0.212	0.29	Hoffmann et al. [8], Warren et al. [26]
*RP4-710M16.1-PPAP2B-PLPP3*	rs112557609	1	56,111,252	A	PP	0.227	0.22	Hoffmann et al. [8], Warren et al. [26]
*RPL34P18-CDH17*	rs7006531	8	94,098,516	G	BP	NA	NR	Liang et al. [35]
*RPL35P4-LOC107986733*	rs10279895	7	27,288,591	G	DBP	0.7553	NR	Liang et al. [35]
*RPL35P4-LOC107986733*	rs11563582	7	27,312,031	A	BP	NA	NR	Liang et al. [35]
*RPL6-PTPN11-ALDH2*	rs11066280	12	112,379,979	T	DBP	1.01	0.04	Kato et al. [22]
*RPS29P9-LOC102724714*	rs3845811	2	207,656,788	G	SBP	0.284	0.43	Wain et al. [27]
*RRAS*	rs61760904	19	49,636,675	T	SBP	1.499	<0.01	Surendran et al. [29]
*RSPO3*	rs13209747	6	126,794,309	T	DBP	0.56	0.35	Franceschini et al. [24]
*RYK*	rs9859176	3	134,281,183	T	SBP	0.322	0.25	Hoffmann et al. [8], Warren et al. [26]
*SBNO1*	rs1060105	12	123,321,672	T	DBP	−0.182	0.18	Surendran et al. [29]
*SCAI-PPP6C*	rs72765298	9	125,138,717	T	PP	−0.374	0.06	Hoffmann et al. [8], Warren et al. [26]
*SDCCAG8*	rs953492	1	243,307,890	A	DBP	0.22	0.49	Hoffmann et al. [8], Warren et al. [26]
*SENP2*	rs12374077	3	185,599,886	C	DBP	0.163	0.42	Hoffmann et al. [8], Warren et al. [26]
*SEPT9*	rs57927100	17	77,321,218	G	SBP	−0.489	0.01	Wain et al. [27]
*SETBP1*	rs12958173	18	44,562,012	A	SBP	0.386	0.25	Ehret et al. [12]
*SH2B3*	rs3184504	12	111,446,804	T	SBP	0.75	0.33	Levy et al. [9], Newton-Cheh et al. [10]
*SLC12A9*	rs7801190	7	100,860,471	C	BP	1.31	0.72	Lettre et al. [39]
*SLC14A2*	rs7236548	18	45,517,785	A	PP	0.352	0.3	Hoffmann et al. [8], Warren et al. [26]
*SLC20A2*	rs2978456	8	42,467,247	T	PP	−0.188	0.45	Hoffmann et al. [8], Warren et al. [26]
*SLC24A3*	rs6081613	20	19,485,263	A	PP	0.263	0.31	Hoffmann et al. [8], Warren et al. [26]
*SLC35F1*	rs9372498	6	118,251,323	A	DBP	0.334	0.07	Hoffmann et al. [8], Warren et al. [26]
*SLC39A8*	rs13107325	4	102,267,552	T	DBP	−0.684	0	Johnson et al. [30]
*SLC4A7*	rs11716531	3	27,415,717	A	DBP	0.213	0.237	Wain et al. [27]
*SLC4A7*	rs13082711	3	27,496,418	T	DBP	−0.238	0.12	Johnson et al. [30]
*SLC8A1*	rs4952611	2	40,340,603	T	DBP	−0.157	0.34	Warren et al. [26]
*SMARCA2-VLDLR*	rs872256	9	2,496,480	T	SBP	0.096	0.43	Parmar et al. [37]
*SNORD32B*	rs926552	6	29,580,312	T	DBP	−0.31	0.07	Liu et al. [23]
*SNX31*	rs2978098	8	100,664,447	A	DBP	0.165	0.34	Warren et al. [26]
*SOX6*	rs4757391	11	16,281,393	C	DBP	0.49	0.28	Lu et al. [51]
*SSPN*	rs6487543	12	26,285,256	A	SBP	0.3	0.46	Warren et al. [26]
*ST7L-CAPZA1-MOV10*	rs2932538	1	112,673,921	G	DBP	0.24	0.17	Johnson et al. [30]
*STK39*	rs6749447	2	168,184,876	G	SBP	3	0.48	Wang et al. [40]
*SUGCT*	rs76206723	7	40,408,372	A	PP	−0.346	0.18	Hoffmann et al. [8], Warren et al. [26]
*SULT1C3*	rs6722745	2	108,258,788	C	SBP	0.28	0.4	Liu et al. [23]
*SVEP1*	rs111245230	9	110,407,495	C	SBP	0.94	0.03	Liu et al. [23]
*SWAP70*	rs2649044	11	9,742,422	T	DBP	0.2	0.547	Wain et al. [27]
*TBC1D1-FLJ13197*	rs2291435	4	38,385,774	T	SBP	−0.378	0.4	Ehret et al. [12]
*TBX5-TBX3*	rs2384550	12	114,914,926	A	DBP	−0.35	0.29	Levy et al. [9], Kato et al. [22]
*TCF7L1*	rs11689667	2	85,264,242	T	PP	0.176	0.28	Hoffmann et al. [8], Warren et al. [26]
*TCF7L2*	rs34872471	10	112,994,312	T	PP	−0.226	0.24	Hoffmann et al. [8]
*TEX41*	rs1438896	2	144,888,505	T	DBP	0.234	0.3	Hoffmann et al. [8], Warren et al. [26]
*TEX41*	rs55944332	2	144,969,054	G	DBP	0.267	0.24	Wain et al. [27]
*TFAP2D*	rs78648104	6	50,715,296	T	SBP	−0.481	0.09	Warren et al. [26]
*TM6SF1*	rs2034618	15	83,130,880	C	DBP	0.21	0.22	Hoffmann et al. [8]
*TMEM161B*	rs10059921	5	88,218,698	T	SBP	−0.526	0.06	Hoffmann et al. [8], Warren et al. [26]
*TMEM194B-NEMP2-NAB1*	rs7592578	2	190,574,865	T	DBP	−0.240	0.18	Hoffmann et al. [8], Warren et al. [26]
*TNRC6A*	rs11639856	16	24,777,324	A	SBP	−0.37	0.17	Liu et al. [23]
*TNRC6B*	rs470113	22	40,333,610	A	PP	−0.253	0.21	Surendran et al. [29]
*TNS1*	rs1063281	2	217,804,009	T	DBP	−0.200	0.43	Hoffmann et al. [8], Warren et al. [26]
*TNXB*	rs2021783	6	32,077,074	C	DBP	0.49	0.79	Lu et al. [51]
*TNXB*	rs185819	6	32,082,290	C	SBP	0.365	0.513	Wain et al. [27]
*TP53-SLC2A4*	rs78378222	17	7,668,434	T	PP	0.904	0	Hoffmann et al. [8], Warren et al. [26]
*TRAPPC9*	rs4288356	8	140,045,627	A	PP	0.224	0.615	Wain et al. [27]
*TRAPPC9*	rs4454254	8	140,049,929	A	PP	−0.261	0.45	Warren et al. [26]
*TRIM36*	rs10077885	5	115,054,424	A	DBP	−0.194	0.42	Ehret et al. [12]
*UBA52P4-LOC105377005*	rs820430	3	27,507,409	A	SBP	0.76	0.32	Lu et al. [51]
*ULK4*	rs7651190	3	41,724,463	G	BP	NA	NR	Liang et al. [35]
*ULK4*	rs9815354	3	41,912,651	A	DBP	0.6	0.17	Levy et al. [9]
*ULK4*	rs7372217	3	41,948,630	G	BP	NA	NR	Liang et al. [35]
*UMOD*	rs13333226	16	20,354,332	NA	HTN	NA	0.24	Padmanabhan et al. [25]
*VAC14*	rs117006983	16	70,721,707	A	PP	0.986	0	Warren et al. [26]
*WNT3A*	rs2760061	1	228,003,374	A	DBP	0.23	0.35	Hoffmann et al. [8], Warren et al. [26]
*XKR6*	rs10107145	8	10,900,703	G	SBP	0.361	0.528	Wain et al. [27]
*XRCC6*	rs73161324	22	41,642,782	T	PP	0.496	0.02	Warren et al. [26]
*ZBTB38*	rs16851397	3	141,415,976	A	DBP	−0.493	0.05	Surendran et al. [29]
*ZC3HC1*	rs11556924	7	130,023,656	T	DBP	−0.214	0.27	Ehret et al. [12]
*ZFAT*	rs894344	8	134,600,502	A	SBP	−0.258	0.47	Warren et al. [26]
*ZNF101*	rs2304130	19	19,678,719	A	DBP	−0.292	0.11	Surendran et al. [29]
*ZNF318-ABCC10*	rs10948071	6	43,312,975	T	PP	−0.38	0.43	Ganesh et al. [34]
*ZNF385B*	rs13407401	2	179,850,979	A	SBP	0.434	0.291	Wain et al. [27]
*ZNF638*	rs3771371	2	71,400,409	T	PP	−0.160	0.37	Hoffmann et al. [8], Warren et al. [26]
*ZNF652*	rs12940887	17	49,325,445	T	DBP	0.26	0.374	Wain et al. [27]
*ZNF652*	rs16948048	17	49,363,104	G	DBP	0.39	0.29	Newton-Cheh et al. [10]
*ZNRF3*	rs4823006	22	29,055,683	G	SBP	−0.33	0.45	Liu et al. [23]

SBP: systolic blood pressure; DBP: diastolic blood pressure; PP: pulse pressure; MAP: mean arterial pressure; HTN: hypertension; NR: not recorded; NA: not available.
